# Automated generation of ground truth images of greenhouse-grown plant shoots using a GAN approach

**DOI:** 10.1186/s13007-025-01441-1

**Published:** 2025-10-04

**Authors:** Sajid Ullah, Narendra Narisetti, Kerstin Neumann, Thomas Altmann, Jan Hejatko, Evgeny Gladilin

**Affiliations:** 1 CEITEC-Central European Institute of Technology, Masaryk University, Brno, Czech Republic; 2 National Centre for Biomolecular Research, Masaryk University, Brno, Czech Republic; 3 Leibniz Institute of Plant Genetics and Crop Plant Research, Gatersleben, Germany

**Keywords:** High-throughput greenhouse imaging, Plant phenotyping, Image segmentation, Ground truth data generation, Deep learning, Generative adversarial network (GAN)

## Abstract

The generation of a large amount of ground truth data is an essential bottleneck for the application of deep learning-based approaches to plant image analysis. In particular, the generation of accurately labeled images of various plant types at different developmental stages from multiple renderings is a laborious task that substantially extends the time required for AI model development and adaptation to new data. Here, generative adversarial networks (GANs) can potentially offer a solution by enabling widely automated synthesis of realistic images of plant and background structures. In this study, we present a two-stage GAN-based approach to generation of pairs of RGB and binary-segmented images of greenhouse-grown plant shoots. In the first stage, FastGAN is applied to augment original RGB images of greenhouse-grown plants using intensity and texture transformations. The augmented data were then employed as additional test sets for a Pix2Pix model trained on a limited set of 2D RGB images and their corresponding binary ground truth segmentation. This two-step approach was evaluated on unseen images of different greenhouse-grown plants. Our experimental results show that the accuracy of GAN predicted binary segmentation ranges between 0.88 and 0.95 in terms of the Dice coefficient. Among several loss functions tested, Sigmoid Loss enables the most efficient model convergence during the training achieving the highest average Dice Coefficient scores of 0.94 and 0.95 for *Arabidopsis* and maize images. This underscores the advantages of employing tailored loss functions for the optimization of model performance.

**Supplementary Information:**

The online version contains supplementary material available at 10.1186/s13007-025-01441-1.

## Introduction

Image-based plant phenotyping is a critical tool for advancing our understanding of plant growth, development, and responses to environmental factors. By enabling the extraction of detailed morphological and physiological traits from plant images, it provides valuable insights that support crop improvement and precision agriculture. However, the first critical step in the quantitative analysis of plant image data is image segmentation, which aims to classify all image pixels into two or more distinctive classes, e.g., foreground (plant) and background (non-plant) regions. Recent reviews and community datasets underscore this impact across crops and imaging modalities, from RGB to hyperspectral and 3D point clouds [1–3].

The absence of efficient ways to generate sufficiently large amounts of ground truth data poses a major bottleneck for the application of contemporary AI approaches to automated plant image segmentation in high-throughput phenotyping of greenhouse-grown plants. In recent times, deep learning algorithms have achieved remarkable success in diverse fields, including computer vision and natural language processing. However, the successful application of deep learning approaches for automated plant image segmentation is contingent upon access to large volumes of high-quality ground truth data. The performance of these models is significantly influenced by the availability of extensive, labeled datasets, which remain a major bottleneck in the automated image analysis workflows for quantitative plant phenotyping [4–6]. Generating accurately segmented reference (i.e., ground truth) images is often labor-intensive and requires substantial time investment, as it involves intricate human–machine interactions for manual or semi-automated annotation and editing [7]. Dataset augmentation has emerged as a common strategy to mitigate this issue, applying artificial transformations such as rotation, scaling, and translation to extend the training dataset. While augmentation technique enhances the size of the dataset, it is inherently restricted by the variability present in the original dataset. For instance, if a specific plant phenotype, such as a plant with seven leaves, is absent in the initial dataset, augmentation cannot introduce such novel instances. Conventional data-augmentation operates exclusively at the pixel level: geometric or photometric transformations—such as rotation, scaling, flipping, or brightness adjustment—modify existing images without expanding the underlying distribution of plant appearances. It only rearranges existing pixels; it cannot introduce genuinely novel phenotypes or lighting regimes, leaving the data-diversity gap unresolved In contrast, generative GANs learn the underlying probability distribution of plant appearances, morphological combinations and can sample entirely new images—introducing phenotypes or illumination conditions never captured by the camera [8, 9].

Generative AI models, including Variational Autoencoders (VAEs) [10] and Diffusion Models [11], enable the synthesis of highly realistic and diverse data, capturing greater natural variations with enhanced fidelity. While VAEs are capable of generating synthetic data, they optimize a reconstruction-based loss function that tends to produce over-smoothed outputs. This characteristic is particularly limiting in tasks like plant morphology segmentation, where preservation of fine details—such as leaf boundaries and texture—is crucial [12]. Generative models were introduced to go beyond pixel-level augmentation: ARIGAN synthesized Arabidopsis rosettes to enrich training sets, and later studies used CycleGAN/StyleGAN variants to transfer styles (e.g., stress symptoms, illumination) or to synthesize novel leaf morphologies and canopy arrangements [13–16]. More broadly, DatasetGAN and BigDatasetGAN showed that GANs can be paired with automatic pixel labeling to scale dataset creation in generic vision, a strategy now being explored for plant imagery [17, 18]. It depends on a heavyweight backbone GAN and a small set of finely annotated images, demanding substantial GPU resources. Very recent work has begun to test diffusion models and controllable pipelines (e.g., ControlNet-guided DDPMs) for plant organs, reporting improved texture fidelity at higher computational cost [19, 20]. Inference with diffusion models is computationally demanding—often requiring several hundred denoising iterations—and, because they do not intrinsically generate pixel-accurate masks, an additional segmentation step is still required. In contrast, GANs, and specifically FastGAN, leverage adversarial loss to produce visually sharper and structurally rich images that better reflect the complexity and variability of real plant structures. Therefore, FastGAN was selected as the preferred method in our study for generating realistic RGB imagery suitable for subsequent semantic annotation tasks. Here we explore the potential of state-of-the-art generative models Pix2Pix and FastGAN to synthesize realistic plant images and their segmented counterparts [21, 22]. These models provide a means of generating diverse and novel datasets that surpass the constraints of traditional augmentation methods. In particular, this feasibility study aims to investigate the efficiency of Generative Adversarial Networks (GANs) by solving the task of automated generation of new or additional ground truth images of greenhouse-grown plant shoots. Our aim is to relieve that manual burden by synthesising ready-to-train RGB–mask pairs using only modest GPU time and a small seed of hand-annotated images. GANs have previously been shown to represent a promising tool for generation of synthetic new data in manyfold applications [13, 23]. By definition, GANs are a type of neural network that learn to generate new data samples that still exhibit a similar basic feature as in the reference training dataset. GANs consist of two components: a generator that produces synthetic data samples and a discriminator that evaluates the authenticity of the generated samples. The generator is trained to produce synthetic samples that are difficult for the discriminator to distinguish from real samples. Through this adversarial training process, the generator becomes more adept at producing synthetic samples that closely resemble the real data.

In this study, we address the challenge of limited annotated data in greenhouse plant phenotyping by proposing a two-stage generative adversarial network-based pipeline that produces pairs of new realistic RGB and binary segmented images of greenhouse-grown plants. In the first stage, we generate independent sets of 2D RGB images of real plants using FastGAN, a GAN-based method that performs non-linear intensity and texture transformation of feature map images. The generated images are used as test images for the second stage. In the second stage, we train a Pix2Pix-based conditional GAN on a small set of 2D RGB real-annotation image pairs. After training, the Pix2Pix network is applied to the synthetic RGB images generated by FastGAN to produce corresponding segmentation masks. To rigorously evaluate the accuracy of these predicted masks, we manually annotated a subset of the FastGAN outputs and computed the per-image Dice coefficient between the Pix2Pix predictions and the manual annotations. We trained the image-annotation generator on three greenhouse-imaged datasets: whole-shoot barley, *Arabidopsis*, and maize. We evaluated the realism of generated images quantitatively with a distribution-similarity metric and qualitatively by inspecting key botanical traits—leaf continuity/attachment, canopy architecture, leaf shape/texture, and the absence of background-imprinting artifacts—on randomly sampled GAN outputs.

## Methods

### Image acquisition

High-resolution images of barley plants (3315 $\times$ 4462 pixels) were acquired at the Institute of Plant Genetics and Crop Plant Research (IPK) using the advanced LemnaTec high-throughput greenhouse phenotyping system. For maize, a direct overhead imaging approach was employed, providing detailed representations of the canopy structure. All images were acquired as 24-bit RGB and saved as PNG. Native resolutions were 3315 $\times$ 4462 px for barley and maize, and 2056 $\times$ 2454 px for Arabidopsis.

#### Data preparation

Before training, we applied basic preprocessing; for Arabidopsis this included minor cropping to remove peripheral non-plant regions. All images were then resized to 1024 $\times$ 1024 px for both FastGAN and Pix2Pix and per-channel normalized to [0, 1].

**Table 1:** Types and views of plant images used in the proposed GAN-based pipeline

Plant	Top view	Side view
*Arabidopsis*	120	**–**
Barley	**–**	300
Maize	120	**–**

**Fig. 1:**
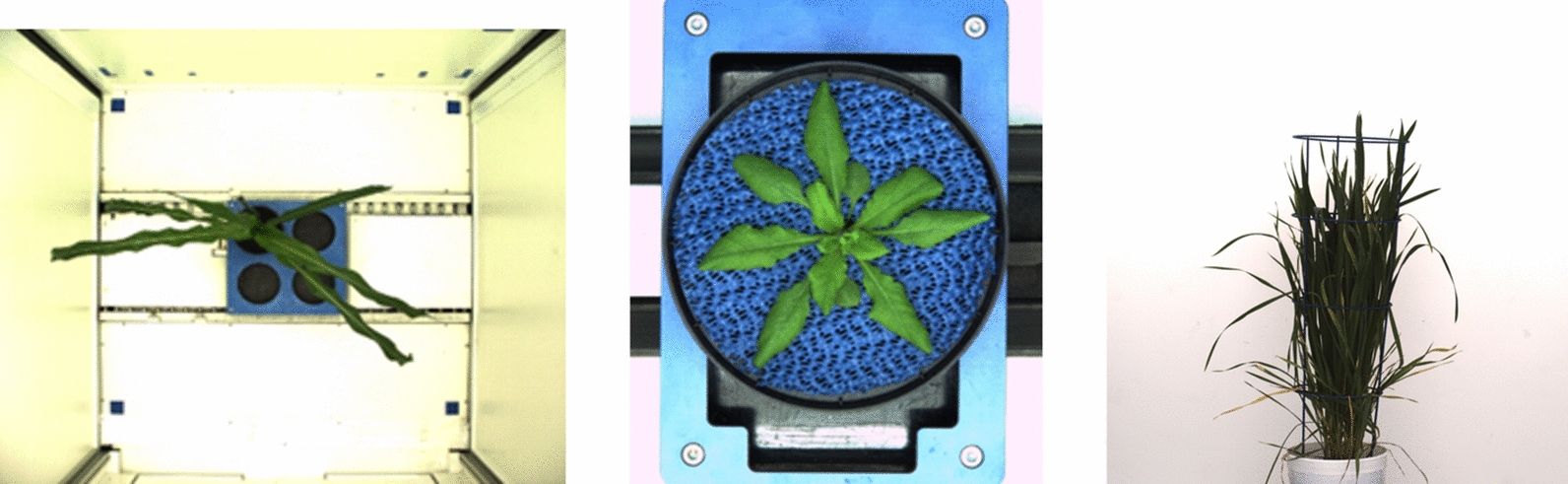
Dataset comprising top-view images of maize and *Arabidopsis*, along with side-view images of barley, acquired using the IPK LemnaTec photo chamber for this study

This perspective is particularly advantageous for quantitative analyses, including leaf area estimation, assessment of plant health, and monitoring developmental stages. Figure 1 presents examples of the images used in this study, including a top-view of maize showcasing its canopy architecture, a top-view of *Arabidopsis*, and a side-view of barley highlighting its structural features.

For training FastGAN, we used 300 barley images and 120 images each of *Arabidopsis* and maize (Table 1) . The synthetic images generated by FastGAN were manually annotated into binary masks using kmSeg [24] and GIMP. Pix2Pix was trained on 100 barley RGB–mask pairs and 80 pairs each for *Arabidopsis* and maize; the test set comprised 25 barley images and 20 images for both *Arabidopsis* and maize, with no overlap with training. The conditional PatchGAN discriminator learns from many overlapping patches per image and, together with the adversarial + L1 losses, provides a strong structural prior that enables effective learning from fewer examples, whereas the purely supervised U-Net relies on pixel-wise losses and therefore benefits from a larger, more diverse labeled set. The original U-Net and its augmented variant were used as baseline models [25]. For *Arabidopsis* and maize (120 images each), we used an 80/20 split (96 train, 24 test). For barley, we trained two U-Net variants on 240 and 300 images, respectively, with 20% held out for testing (48 and 60 images) to probe data-size sensitivity and approximate the minimal effective training set. The U-Net was implemented and trained as a supervised baseline model. Its primary role is to serve as a point of comparison for evaluating the segmentation performance of our proposed Pix2Pix-based pipeline. Table 2 summarizes the training and test set sizes as well as the top- and side-view image counts for each model and plant species. Barley required a larger set than *Arabidopsis* and maize because side-view whole-shoot segmentation shows greater morphological and pose variability (e.g., tillering, leaf overlap). No data augmentation was applied during Pix2Pix training; instead, we relied on the PatchGAN discriminator’s patch diversity.

**Table 2:** Training and test set sizes for FastGAN, Pix2Pix, and U-Net models for three plant species, including the view type of images used

Model	Plant	Training set size	**Test set size**	**View type**
FastGAN [26]	barley	300	–	Side
	*Arabidopsis*	120	–	Top
	Maize	120	–	Top
Pix2Pix [22]	barley	100 RGB-mask pairs	25	Side
	*Arabidopsis*	80 RGB-mask pairs	20	Top
	Maize	80 RGB-mask pairs	20	Top
U-Net [25]	barley (variant 1:min)	240	48	Side
	Barley (variant 2:max)	300	60	Side
	*Arabidopsis*	96	24	Top
	Maize	96	24	Top

### Generative adversarial networks

#### FastGAN

FastGAN learns an unconditional GAN on high-resolution images. We incorporate a skip-layer channel-wise excitation (SLE) module to fuse multi-scale information. Specifically, low-resolution activation maps are globally pooled and passed through a two-layer MLP to generate channel-wise weights, which are then used to recalibrate the high-resolution feature maps. This mechanism enables coarse contextual cues to selectively enhance fine-scale details [21]. SLE allows a more robust gradient flow throughout the model weights for faster training. The self-supervised discriminator *D* is trained as a feature-encoder with an extra decoder. Let $F_\ell \in \mathbb {R}^{C\times H_\ell \times W_\ell }$ be a low-resolution feature map and $F_h\in \mathbb {R}^{C\times H_h\times W_h}$ its high-resolution counterpart. We first obtain a channel descriptor via global average pooling:

1 $$\begin{aligned} z_c \;=\;\frac{1}{H_\ell W_\ell }\sum _{i=1}^{H_\ell }\sum _{j=1}^{W_\ell }F_\ell [c,i,j]\quad (c=1,\dots ,C). \end{aligned}$$

This vector is passed through a two-layer MLP (with weight matrices $W_1,W_2$ and activations ReLU and sigmoid) to yield channel weights

2 $$\begin{aligned} a = \sigma \bigl (W_2\,\textrm{ReLU}(W_1\,z)\bigr )\;\in (0,1)^C. \end{aligned}$$

Finally, we reshape *a* to $C\times 1\times 1$, broadcast it over $H_h\times W_h$, and apply it to the high-res map:

3 $$\begin{aligned} \widetilde{F}_h[c,i,j] \;=\;a[c]\times F_h[c,i,j], \quad i=1,\dots ,H_h,\;j=1,\dots ,W_h. \end{aligned}$$

We employ a single convolutional layer per resolution in both the generator (G) and discriminator (D), limiting high-resolution ($\ge 512\times 512$) layers to three channels. To generalize skip-connections across scales, we introduce the SLE module. Traditional ResBlocks fuse features via element-wise addition—requiring identical spatial dimensions—whereas our SLE uses channel-wise multiplications, substantially reducing computation. Concretely, we first global-pool the low-res map (Eq. 1), compute channel attention via a two-layer MLP (Eq. 2), then broadcast and apply these weights to the high-res map (Eq. 3). This design preserves ResBlock’s shortcut gradient flow and lightweight operations, while safely fusing multi-scale features without expensive spatial alignment.

#### Conditional generative adversarial networks

Conditional GANs aim to generate data samples (e.g., images) that resemble real data and adhere to specific conditions or constraints. We used the Pix2Pix framework for image-to-image translation, mapping RGB input images to binary segmentation masks [22]. Conditional GANs have two main components: a generator (G) and a discriminator (D). The generator takes as input a random noise vector, drawn from a Gaussian distribution and additional conditional information, often represented as a label vector or other auxiliary data. On the other hand, the discriminator evaluates the realism of generated samples and considers the provided conditional information to make its prediction.

**Fig. 2:**
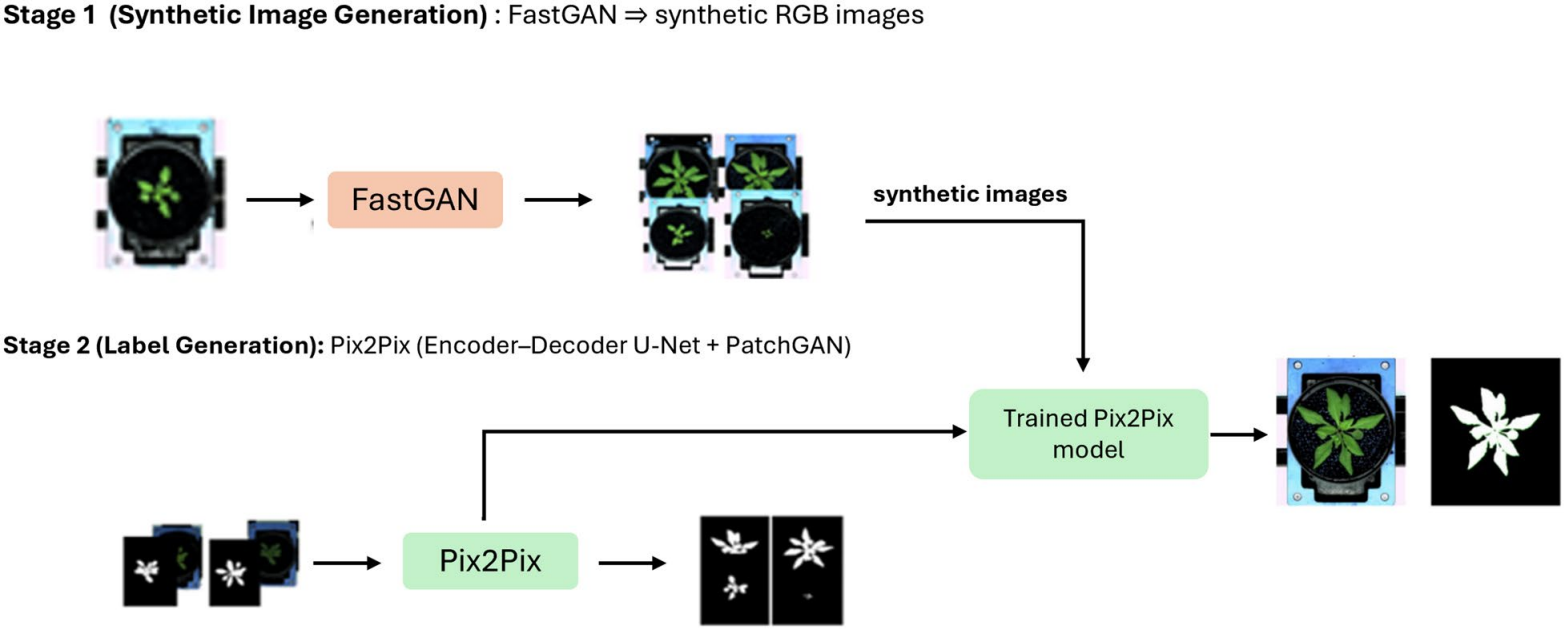
Overview of our annotation pipeline. Stage 1: FastGAN is trained on unlabelled, real high-resolution plant images to produce diverse synthetic RGB renderings (Wang et al., 2018). Stage 2: A Pix2Pix conditional GAN (encoder–decoder U-Net with PatchGAN discriminator) is trained on a small, manually annotated set of real RGB–mask pairs, then applied to the FastGAN outputs to generate pixel-accurate semantic labels. Finally, we evaluate these predicted labels against hand-annotated masks of a held-out synthetic subset to quantify fidelity and guide further refinements

The process of annotation involves two stages. The first stage focuses on generating synthetic images using FastGAN, a generative model designed to produce high-quality images. In this stage, RGB images were acquired from IPK phenotyping facilities, encompassing three datasets: barley, *Arabidopsis*, and maize. The primary objective is to generate synthetic images that closely resemble the original dataset, ensuring that the synthetic images do not deviate significantly in quality or distribution. Additionally, the aim is to produce binary annotations for these images to support segmentation tasks in deep neural networks (DNNs). To achieve this, we tested several cost functions, including binary cross-entropy, Wasserstein loss [27], hinge loss [28], and adversarial loss [29].

In the second stage, we train a Pix2Pix conditional GAN on a compact, manually annotated set of real RGB–mask pairs to learn the mapping from raw images to binary segmentations. Once trained, this network is applied to the synthetic RGB images produced by FastGAN, yielding predicted masks for each synthetic sample. To rigorously assess the fidelity of these predictions, we manually annotate a held-out subset of the FastGAN outputs and compute per-image Dice coefficient between the Pix2Pix predictions and our manual labels. This quantitative evaluation not only validates the realism of the generated ground truths but also provides concrete guidance for refining the upstream synthetic data generator. The annotation pipeline, illustrated in Fig. 2, demonstrates the process of generating synthetic images and their corresponding semantic labels.

### Evaluation of GANs

We used the FID score to assess the quality of the synthetic images generated in the first stage [30]. The FID score measures the similarity between the generated images and the real images. We found that patches generated with FastGAN models yielded the lowest FID scores, indicating that they closely resemble real images. Lower scores indicate the two groups of images are more similar, or have more similar statistics, with a perfect score being 0.0 indicating that the two groups of images are identical. In the second stage, we evaluated the quality of the annotated images generated using a dice coefficient, a measure of overlap between the generated and real images. We also compared the performance of our approach using different cost function for the conditional GANs. Our results demonstrate that our proposed approach can generate realistic 2D patches of plant regions and labels for semantic label segmentation. To accelerate training without loss of segmentation accuracy measured by the average Dice coefficient, we employed PyTorch’s automatic mixed-precision (AMP) on both FastGAN and Pix2Pix, using FP16 for matrix multiplications and FP32 for accumulation. This reduced GPU memory usage by $\sim 30\%$ and halved training time, with no measurable impact on the final Dice scores. The synthetic images generated by FastGAN were evaluated using the FID score. The FID score is calculated using the following formula.

4 $$\begin{aligned} \textrm{FID} = \Vert \mu _r - \mu _g\Vert ^2 + \textrm{Tr}\bigl (\Sigma _r + \Sigma _g - 2(\Sigma _r \Sigma _g)^{1/2}\bigr ), \end{aligned}$$

where

$$ \begin{aligned} \mu _r,\ \Sigma _r&\quad \text {are the mean and covariance of the real images' feature embeddings},\\ \mu _g,\ \Sigma _g&\quad \text {are the mean and covariance of the generated images' feature embeddings},\\ \Vert \cdot \Vert&\quad \text {denotes the Euclidean norm},\\ \textrm{Tr}(\cdot )&\quad \text {is the trace operator}. \end{aligned} $$

Dai et al. [31] highlight the importance of leveraging visual cues to enhance the interpretability and quality of GAN-generated images. For Pix2Pix and U-Net, we use the aDC for evaluation, as defined in Eq. (5). The aDC quantifies the overlap between predicted and ground truth segmentations. For a single image, let $G=\{g_i\}_{i=1}^N$ and $P=\{p_i\}_{i=1}^N$ be the ground-truth and predicted binary masks, respectively, each containing *N* pixels. We obtain *P* by thresholding the network’s per-pixel probability map at 0.5. We then define:

$$ \begin{aligned} \textrm{TP}&= \sum _{i=1}^N \bigl [p_i = 1 \wedge g_i = 1\bigr ],\\ \textrm{FP} & = \sum _{i=1}^N \bigl [p_i = 1 \wedge g_i = 0\bigr ],\\ \textrm{FN} & = \sum _{i=1}^N \bigl [p_i = 0 \wedge g_i = 1\bigr ],\\ \textrm{TN} & = \sum _{i=1}^N \bigl [p_i = 0 \wedge g_i = 0\bigr ]. \end{aligned} $$

where *TP*: True Positives; *FP*: False Positives; *FN*: False Negatives; *N*: Total number of images in the dataset;

The average Dice coefficient (aDC) is defined as:

5 $$\begin{aligned} { \textrm{aDC} = \frac{1}{M}\sum _{j=1}^M \frac{2\,\textrm{TP}_j}{2\,\textrm{TP}_j + \textrm{FP}_j + \textrm{FN}_j} }\,. \end{aligned}$$

where *M* is the number of test masks and $(\textrm{TP}_j,\textrm{FP}_j,\textrm{FN}_j)$ are the pixel counts for the *j*th image. In other words, all four categories (TP/FP/FN/TN) are counted pixel-wise, not image-wise, and the aDC is the mean of per-image Dice scores. We experimented with thresholds $\tau \in \{0.3, 0.4, 0.5, 0.6, 0.7\}$. The macro-averaged $\text {aDC}$ varied by $\le 0.005$ across this range, so we fixed $\tau = 0.5$ for reproducibility.

## Experimental results

This section presents the results of evaluating FastGAN, a generative adversarial network, on various image generation tasks and Pix2Pix. The primary focus was on assessing the model’s efficiency, image quality, and scalability across different datasets.

We used three datasets for our evaluation: *Arabidopsis*, barley, and maize. FastGAN underwent training for 100,000 iterations for each dataset on an NVIDIA RTX 4090 GPU. The training utilized the Adam optimizer, setting the learning rate to 0.0002. Standard GAN loss function was adopted, and spectral normalization was implemented in both the generator and discriminator as a means of regularization. In Pix2Pix, the standard adversarial loss is combined with L1 loss to ensure that the generated image is not only realistic but also closely resembles the ground truth.

The performance of FastGAN was quantitatively analyzed using the FID. The FID measures the distance between the distribution of generated images and real images. One of the key strengths of FastGAN was its efficiency. The training time was significantly lower compared to traditional GAN models without compromising on the quality of the generated images [32]. Additionally, the model scaled well across different datasets, maintaining consistent performance irrespective of the complexity of the dataset. Overall, FastGAN demonstrated its capability as a powerful tool for image generation tasks. It offers a balance between efficiency, image quality, and scalability, making it suitable for various applications in image synthesis.

### Results of barley shoot analysis

**Fig. 3:**
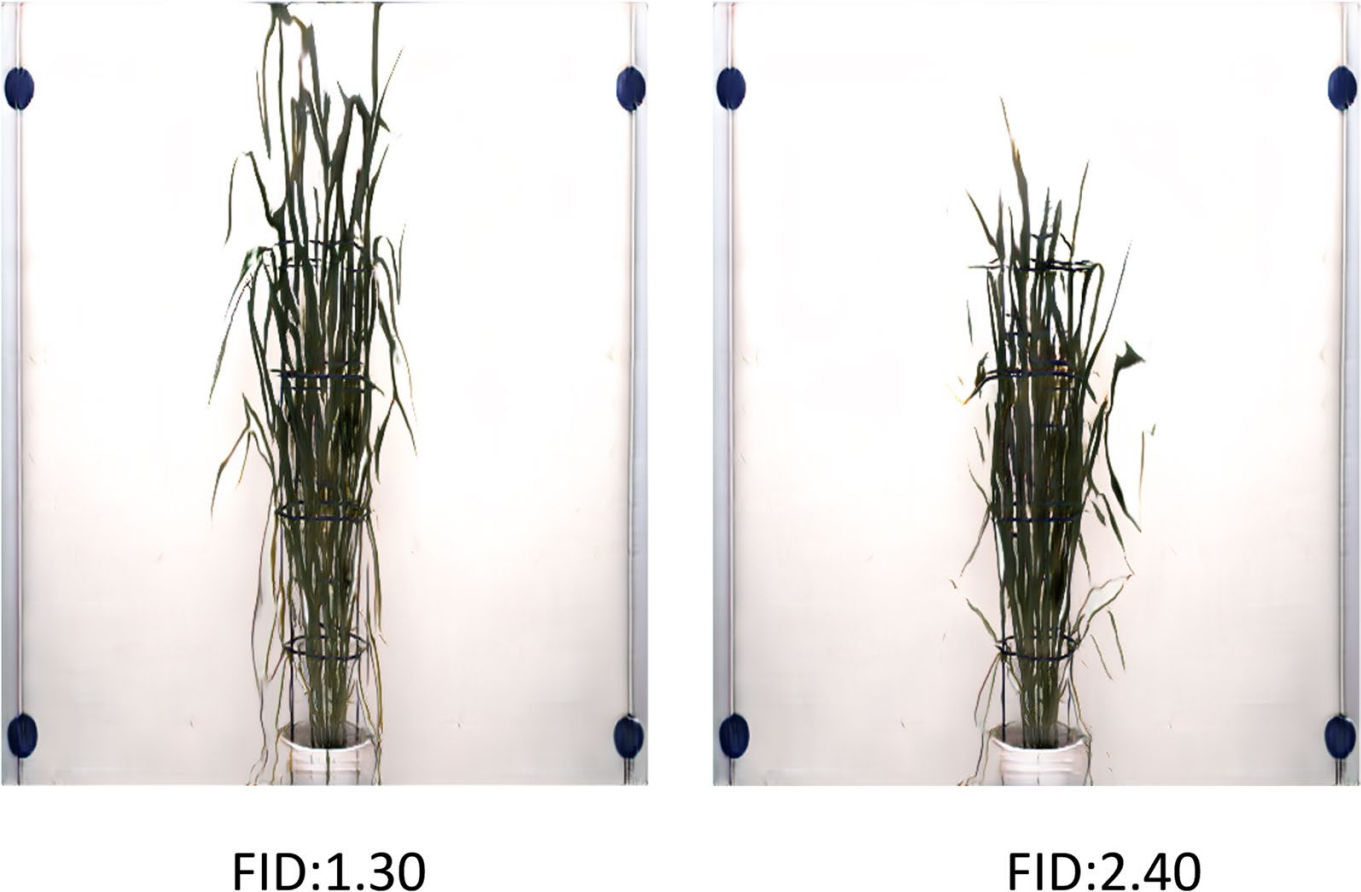
FastGAN-generated images of barley shoots exhibiting varying FID values. The barley shoot on the right shows artifacts, with a leaf appearing disassociated or ‘floating’

The study investigated the generation of synthetic barley shoot images using FastGAN, achieving FID scores of 1.30 and 1.35, which reflect the quality and diversity of the generated outputs. Such scores reflect a modest degree of likeness to the authentic barley shoot imagery. While these FID scores are not remarkably low, they imply that FastGAN successfully replicated key characteristics of the barley shoots. Nonetheless, there is potential for enhancement in diminishing the FID values further to attain a higher fidelity to the original images. These images, shown in Fig. 3, illustrate how the FID values affect the visual realism and diversity of the generated outputs.

In the results of our study on IPK whole shoot segmentation in barley, two deep learning models, CGAN and U-Net, were compared (Table 3).

**Table 3:** Performance comparison between Pix2Pix and U-Net models

Dataset	Pix2Pix	U-Net
Whole barley shoot	0.96	0.95

**Fig. 4:**
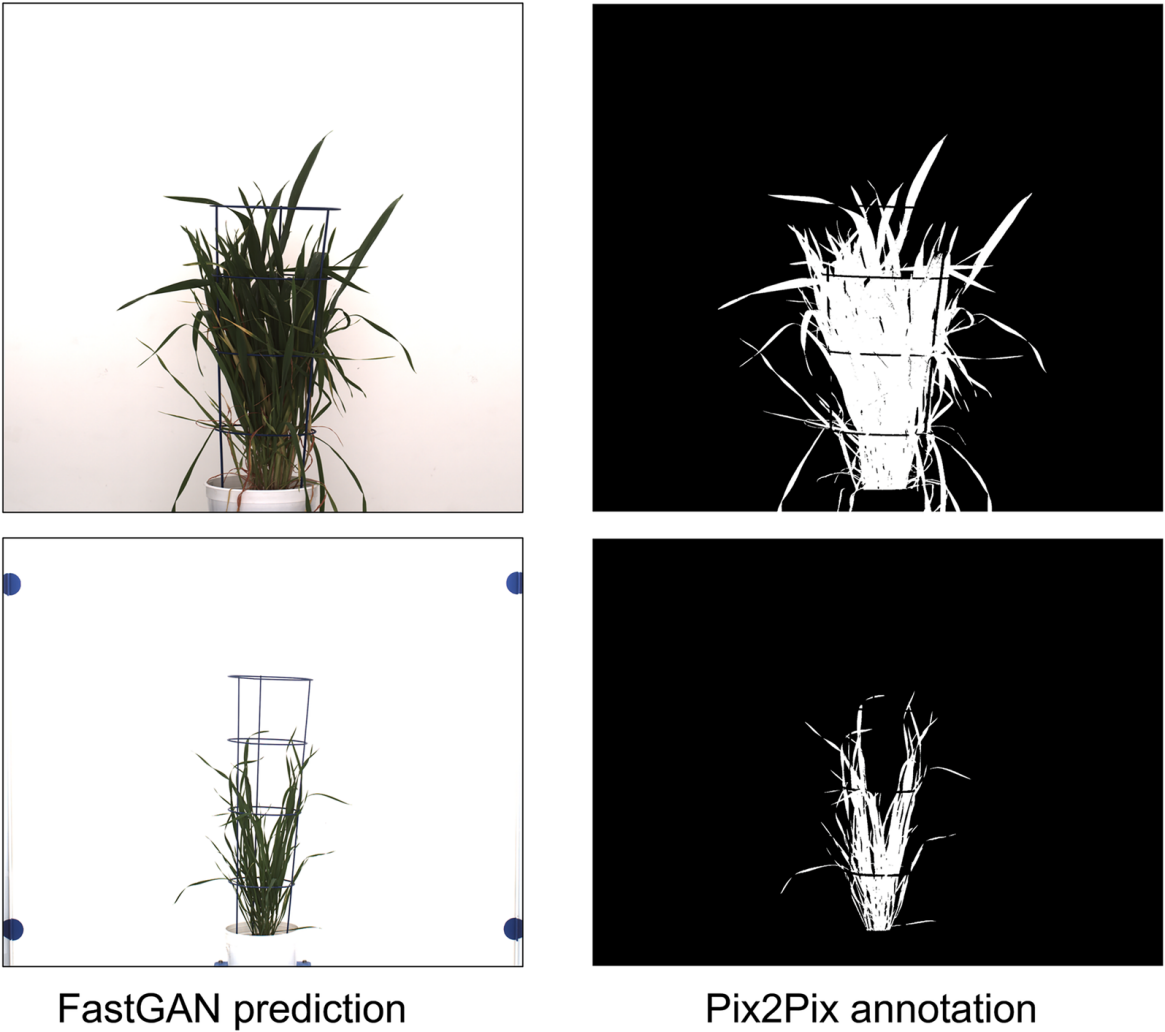
FastGAN-generated image and Pix2Pix binary annotation of barley shoots

The CGAN model exhibited a marginally superior aDC score, registering at 0.96, whereas the U-Net model attained a score of 0.95. This outcome suggests that both models are proficient in executing the segmentation task, with Pix2Pix demonstrating a slight advantage over U-Net, as depicted in Fig. 4. The findings underscore the efficacy of both CGAN and U-Net in the domain of precise agricultural image segmentation, with each model providing strong performance in this particular context.

### Result of Arabidopsis shoot analysis

**Fig. 5:**
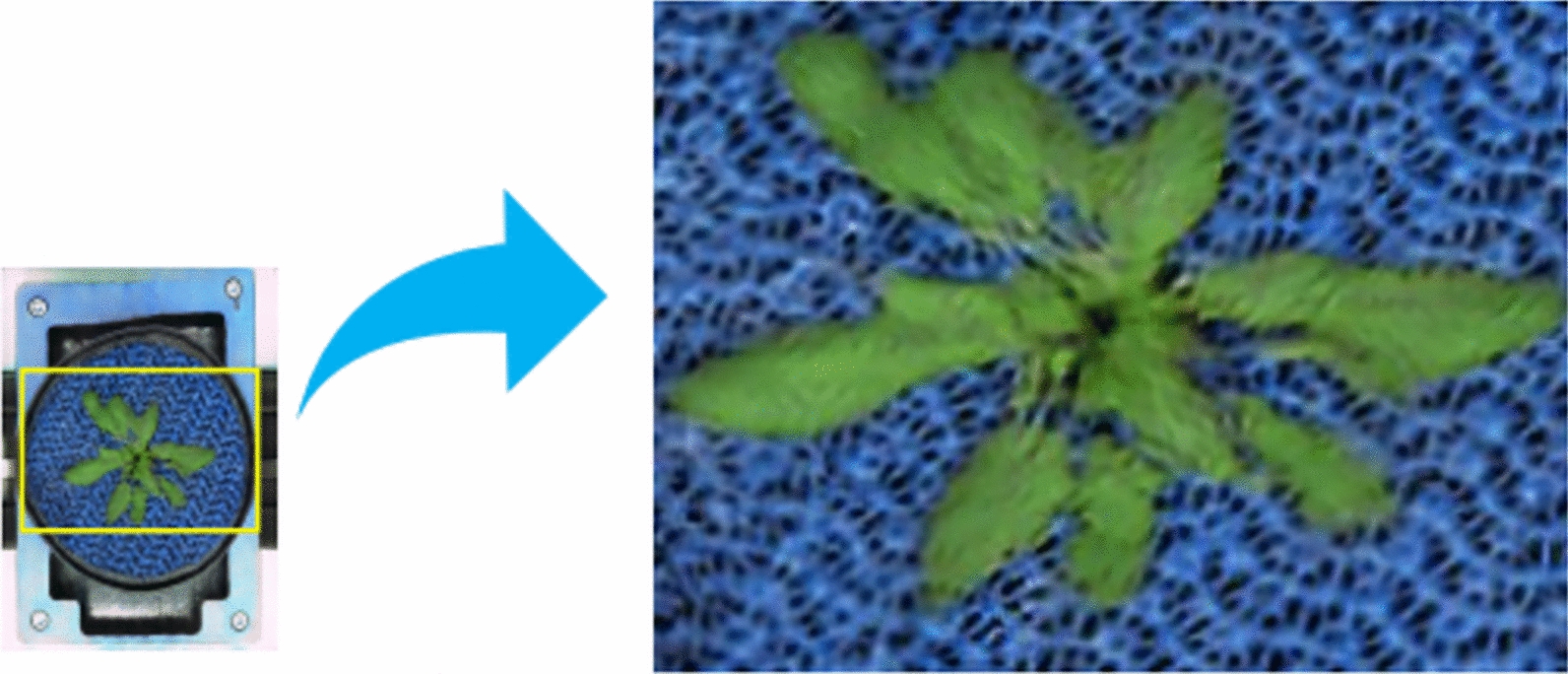
Contextual influence: the variable appearance of blue mats (mostly their relative rotation) in the training images was taken over by the model into representation of plant structures, resulting in the imprinting of ‘wavy’ mat patterns on the *Arabidopsis* shoot

Different from all other plant setups, top-view images of the Arabidopsis plant exhibit high variation in the background areas: the blue mat beneath the plant is not static but varies in its optical appearance (mostly due to the relative rotation of the mat). Consequently, FastGAN predictions of the *Arabidopsis* shoots exhibit an overlay of the ’wavy’ mat pattern with the plant green leaves of the plant shoot, see Fig. 5.

**Fig. 6:**
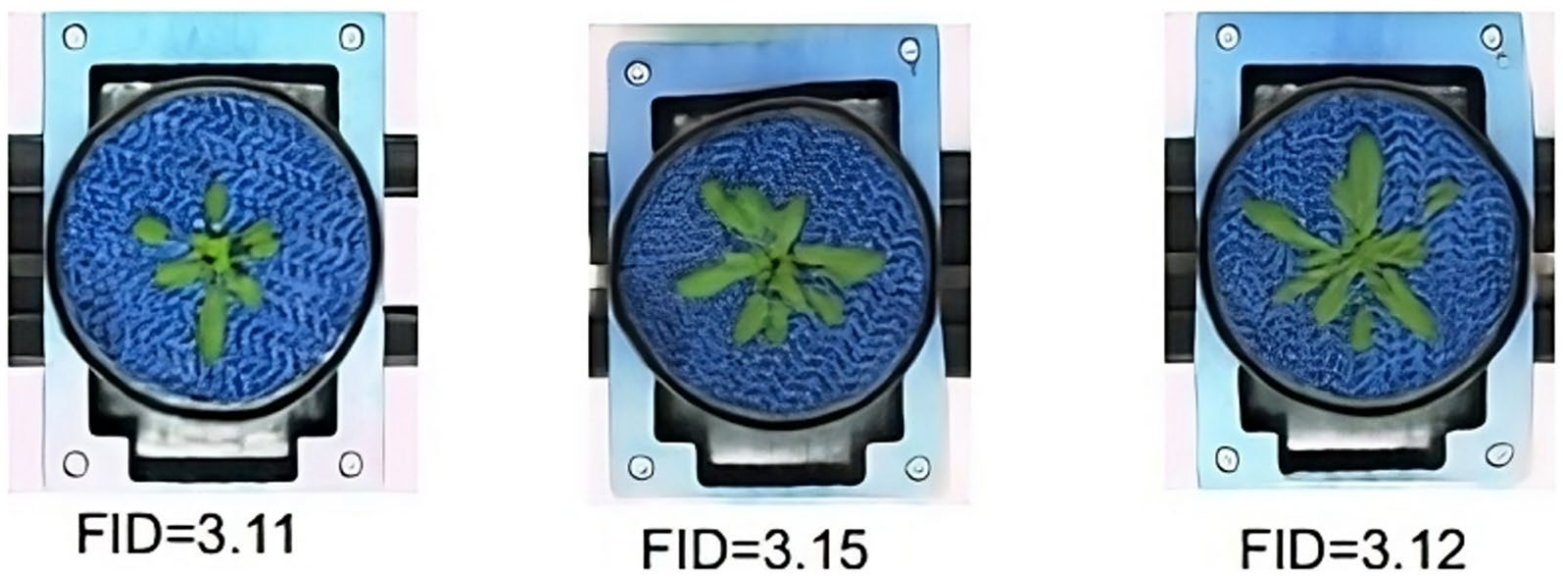
*Arabidopsis* images synthesized using FastGAN, displaying varying FID scores. Lower scores denote a higher resemblance to the original, acquired images

**Fig. 7:**
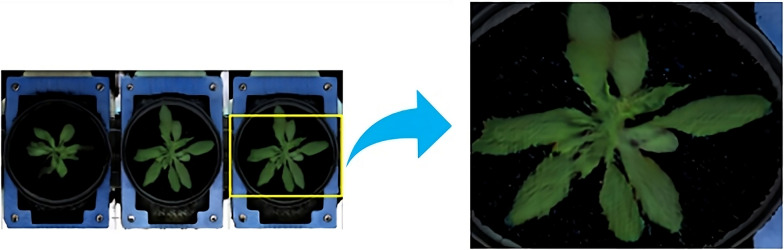
*Arabidopsis* images after removal of the background blue mat

As shown in Fig. 6, lower FID scores indicate higher similarity to the original dataset, emphasizing the effectiveness of the generative process. As a consequence, not only the plants but also background structures exhibit substantial variability in optical appearance, which affects FastGAN’s prediction results. The FastGAN predictions for the original images in the *Arabidopsis* domain exhibit a distinct *mesh pattern* characteristic of the blue mats. This pattern suggests that the GAN consolidates all variable structures into a single category, failing to distinguish effectively between plant and non-plant regions. To dissect the impact of variable background structures, analysis was performed with original *Arabidopsis* images as well as images where those variable blue mats were removed. After removing the background blue mat, as depicted in Fig. 7, the shoot becomes more distinguishable, enabling a clearer analysis of its structure. In the kmSeg tool [24], a semi-automated segmentation approach was employed to preprocess the images. The tool allows for the selection of specific color regions, such as the blue background commonly found in *Arabidopsis* image datasets. The interference regions are selected to be excluded. Using this functionality, the mesh pattern of blue mats was isolated and subsequently removed, resulting in images where the plant regions were cleanly segmented. This preprocessing step ensured that non-plant structures, including the ’wavy’ blue mats, did not interfere with downstream analyses and facilitated the accurate evaluation of plant features. This preprocessing enhanced our dataset’s quality and significantly improved the performance of the downstream image analysis.

Our investigation reveals a marked improvement in FID scores between two datasets of generated images. The unprocessed training set recorded an average FID score of 5, while the mat-removed dataset achieved a significantly lower average of 2.6, indicating a closer resemblance to the original *Arabidopsis* shoot images. This result highlights the GAN’s proficiency in accurately synthesizing the target dataset, demonstrating effective learning and image generation.

Conversely, the second batch of images, which underwent preprocessing for background removal, exhibited a higher aDC score of 0.94, representing a 27% increase compared to the unprocessed images, which had an aDC score of 0.740 as summarized in Table 4. This decrease in FID indicates that eliminating background components improved the visual realism of the generated images, bringing them closer to the original dataset distribution. Although the intention behind preprocessing was to more distinctly isolate the shoots for improved segmentation, this step inadvertently impacted the GAN’s capacity to accurately duplicate the specific features of the original images as achieved with the non-preprocessed set.

**Table 4:** Average Dice coefficient (aDC) for *Arabidopsis* annotation

*Arabidopsis* dataset	aDC
Original (no preprocessing)	0.75
Background removed	0.94

**Fig. 8:**
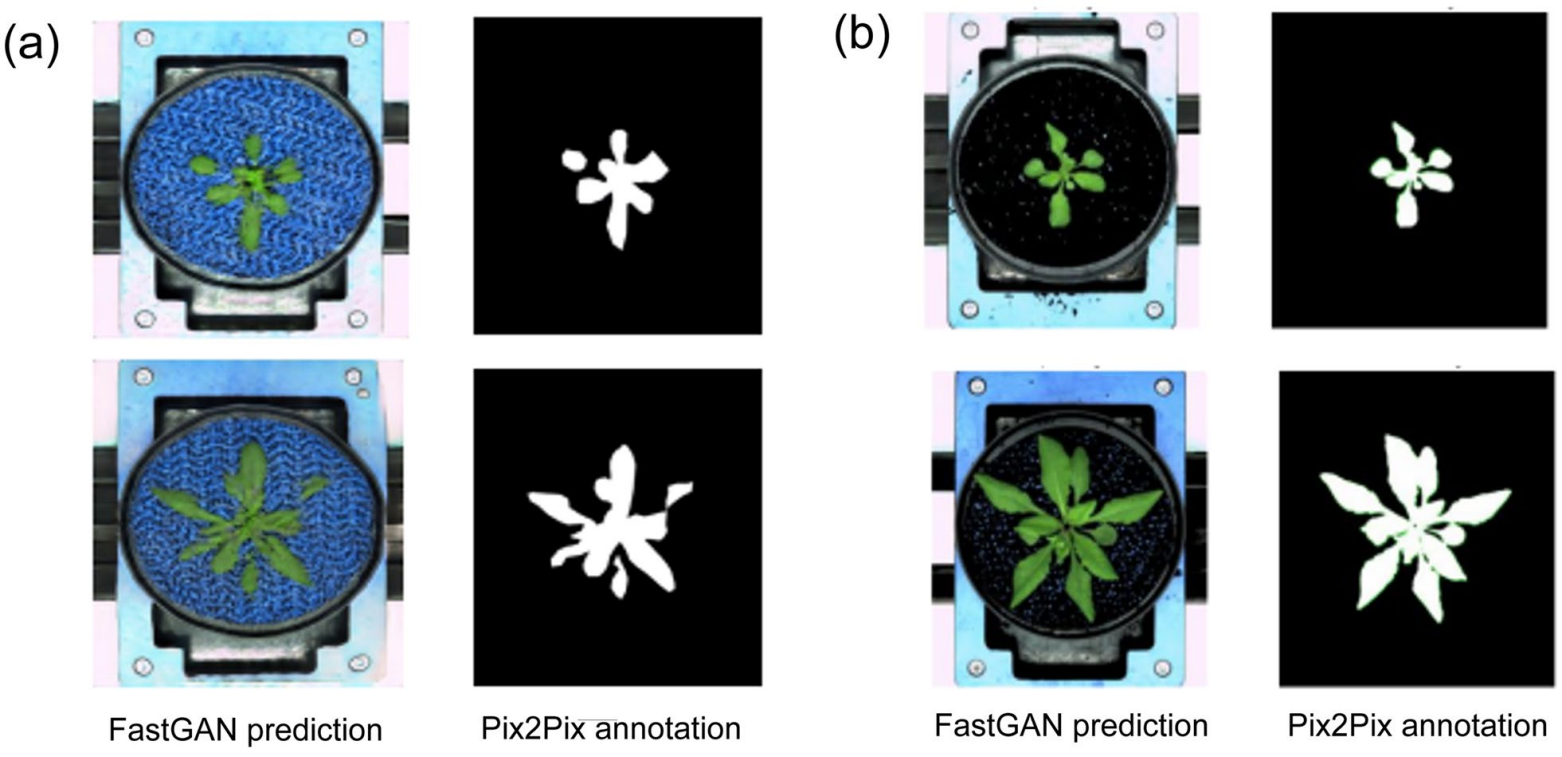
Pix2Pix segmentation of FastGAN generated images: **a** without vs. **b** with background removal

As shown in Fig. 8a, the Pix2Pix results for FastGAN-generated images without background removal illustrate the segmentation accuracy measured by the aDC under unprocessed conditions.

The results depicted in Fig. 8b are noteworthy because they highlight the intricate relationship between pre-processing methods and the fidelity of generated images in machine learning contexts. The increase in the aDC score following preprocessing emphasizes the challenges in attaining ideal image generation, particularly when alterations are applied to the original dataset.

This aspect of the research contributes to a deeper understanding of how preprocessing steps can influence the performance of GANs in image synthesis tasks, particularly in the field of plant phenotyping.

### Results of maize shoot analysis

**Fig. 9:**
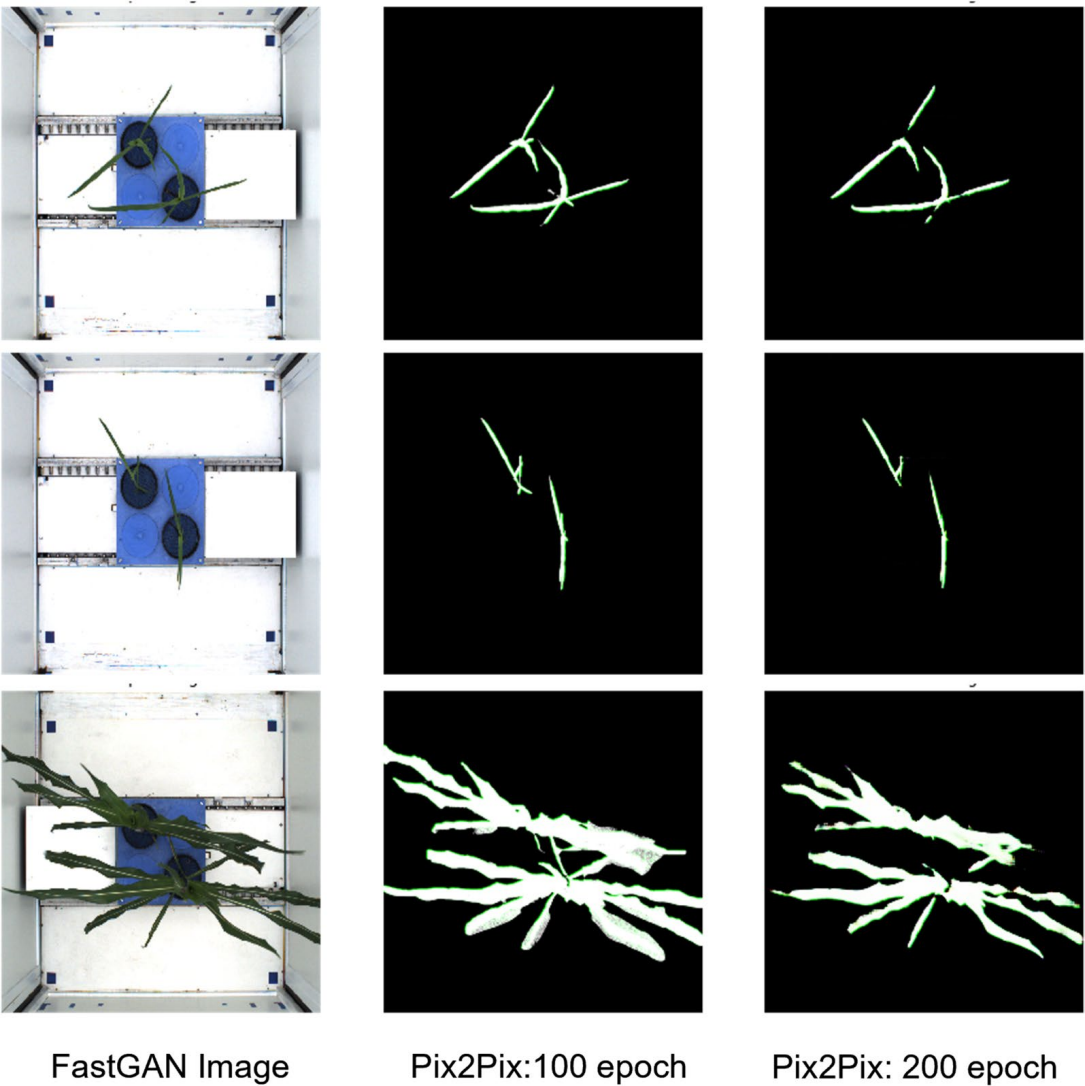
Pix2Pix prediction of FastGAN-generated images after epochs 100 and 200

The training of the Pix2Pix model was conducted across a spectrum of epochs, ranging from 25 to 200. The performance of the model during the initial 50 epochs was suboptimal, primarily attributable to the model’s difficulty with the white background present in the images. This early challenge reflects the Pix2Pix model’s susceptibility to variations in background within the training dataset. Notwithstanding, beyond the 50th epoch, a noticeable enhancement in accuracy was evident, suggesting the model’s incremental acclimatization to the peculiarities of the dataset. Such improvement accentuates the necessity for prolonged training periods when dealing with complex imagery in the realm of deep learning. The Pix2Pix predictions for FastGAN-generated images, as shown in Fig. 9, demonstrate the progressive improvement in output quality between epochs 100 and 200.

### Summary of shoot analysis

As shown in Table 5, Pix2Pix exceeds U-Net performance by 1, 9 and 11 percentage points on barley, *Arabidopsis* and maize, respectively. Moreover, elevating the Pix2Pix input resolution from $512\times 512$ to $1024\times 1024$ drives additional aDC gains of 2, 3 and 5 percentage points (mean$\approx 3.3$pp) across the same datasets. This reinforces the advantage of adversarial methods in scenarios requiring precise boundary delineation, especially for datasets with structural complexity.

Future research should explore hybrid architectures combining the robustness of U-Net’s skip connections with the discriminative learning capacity of GANs. Additionally, domain-specific preprocessing techniques, such as background removal and augmentation strategies, may further enhance U-Net’s performance for plant segmentation tasks.

**Table 5:** Average Dice coefficient for Pix2Pix at two input resolutions (512 $\times$ 512 and 1024 $\times$ 1024) and U-Net at 1024 $\times$ 1024 across all plant datasets. The aDC was computed by comparing manually annotated synthetic images—labelled using kmSeg and GIMP—with the segmentation outputs of the Pix2Pix and U-Net models

Dataset	Pix2Pix ($512^2$)	Pix2Pix ($1024^2$)	U-Net ($1024^2$)
Barley	0.94	0.96	0.95
*Arabidopsis*	0.91	0.94	0.85
Maize	0.90	0.95	0.84

**Fig. 10:**
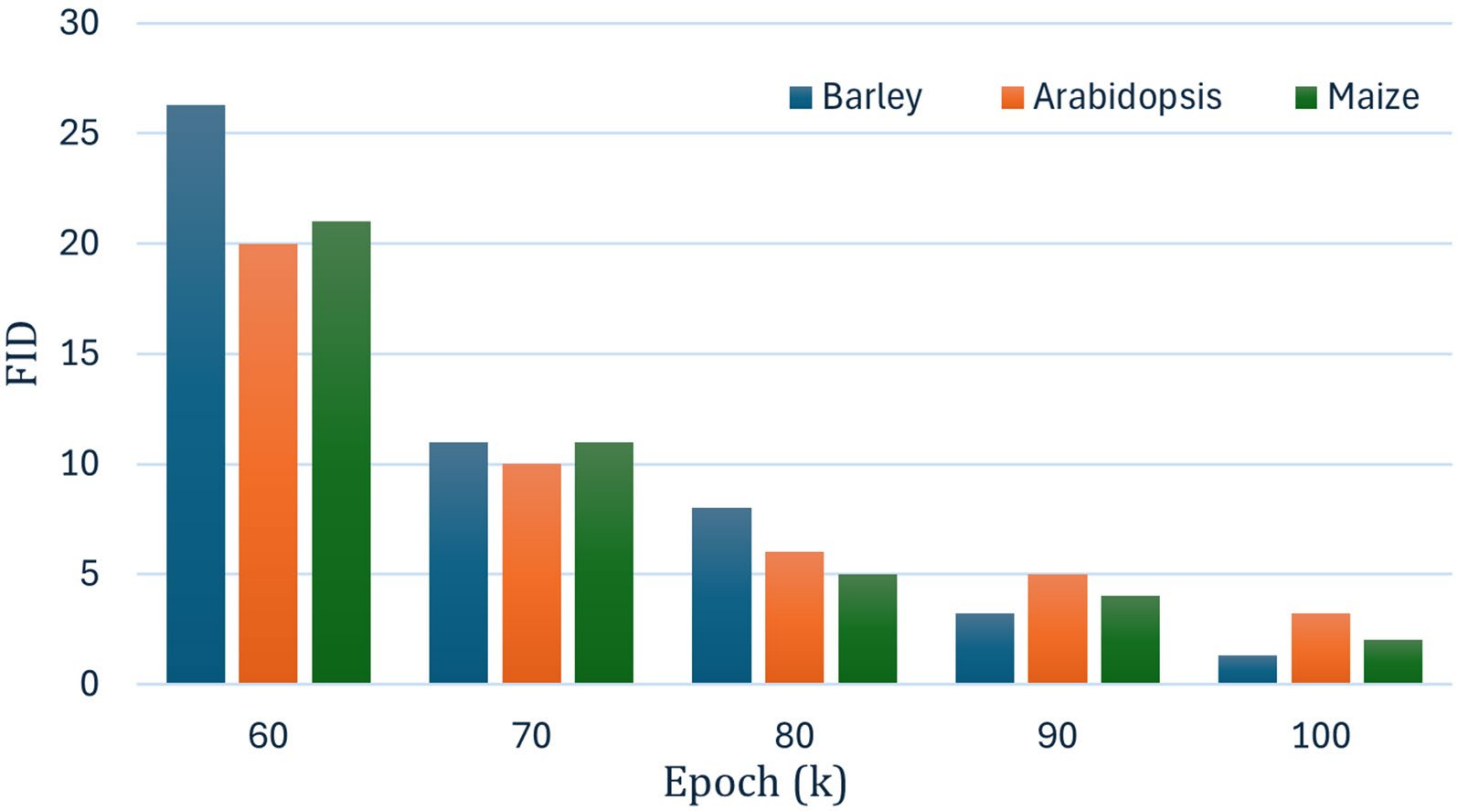
Comparison of FID scores for Barley, *Arabidopsis*, and Maize across different epochs (k)

As shown in Fig. 10, across all datasets, the FID scores exhibit a steady decline as the number of epochs increases, indicating improved image quality and fidelity with extended training. The consistent reduction in FID highlights the model’s ability to generate higher-quality images over time, as it effectively learns the data distribution for barley, *Arabidopsis*, and maize.

**Table 6:** Ablation of Pix2Pix loss functions on *Arabidopsis*, maize and barley (50 epochs)

Loss	aDC		
	*Arabidopsis*	Maize	Barley
Adversarial (AL)	0.90	-–	0.90
Hinge (HL)	0.935	–-	–-
Wasserstein (WL)	0.94	-–	-–
Sigmoid	**0.94**	**0.95**	**0.96**

To provide a conventional baseline, we also trained U-Net with standard on-the-fly augmentations (random flips, $\pm 15^\circ$ rotations, 0.9–1.1 scaling, and mild brightness/contrast jitter). U-Net + augmentation improved Dice by ${<}1\%$ across all datasets.

For the annotation tasks, Pix2Pix was employed to evaluate the *Arabidopsis* and Maize datasets on different cost functions. The *Arabidopsis* dataset achieved aDC of 0.90, 0.935, and 0.94 using Adversarial Loss (AL), Hinge Loss (HL), and Wasserstein Loss (WL), respectively, after 50 epochs, with the highest aDC of 0.94 obtained using Sigmoid Loss, see Table 6. Training time for the *Arabidopsis* dataset averaged 30 min for 50 epochs. Similarly, the Maize dataset achieved an aDC of 0.95 with Sigmoid Loss, while other loss functions, such as AL, HL, and WL, failed to converge or perform segmentation effectively.

### Diversity analysis and mode collapse evaluation

We evaluated Pix2Pix performance (aDC) as a function of training set size for Arabidopsis, barley, and maize. As shown in Supplementary Figure S1, Dice scores increased up to  100 training images, but gains beyond that were marginal. This suggests that Pix2Pix achieves near-optimal performance with relatively small, high-quality training sets. Supplementary Figures S2–S4 show overlays of Pix2Pix-predicted masks for synthetically generated example images of barley, Arabidopsis, and maize shoots. To investigate the feature diversity of GAN-generated shoot images and detect signs of potential mode collapse, we performed t-SNE analysis using 128-dimensional feature embeddings extracted from a ResNet-18 model [33, 34]. For each plant species (*Arabidopsis*, barley, Maize), 100 real and 100 synthetic images from FastGAN were projected into a 2D space. The resulting clusters shown in Supplementary Figures S5–S7 indicate that synthetic samples form well-distributed groups, partially overlapping with real counterparts. The dispersion and spatial distribution of synthetic images suggest that the generator learned diverse mappings rather than collapsing to limited modes. No evidence of severe mode collapse (i.e., tightly packed or highly redundant clusters) was observed.

## Discussion

In this paper, we dealt with generating and annotating synthetic ground truth images of greenhouse-grown plant shoots using a two stage (i.e. FastGAN and Pix2Pix) modeling approach. Our investigations elucidate the capabilities of FastGAN and Pix2Pix in creating synthetic images tailored to a particular plant type and imaging facility. The GAN model’s proficiency in fabricating novel and varied datasets is invaluable, offering significant benefits in areas where data acquisition is traditionally laborious and time-intensive. The FastGAN-generated dataset currently consists of approximately a hundred unique images, beyond which repetitions of familiar plant structures and patterns begin to emerge. While this study generated 100 synthetic images per class, the approach demonstrates a pathway toward scalable data augmentation. We acknowledge that with limited training data, GANs are susceptible to mode collapse—where the model may memorize a few patterns and fail to generalize—resulting in outputs that lack diversity and realism, particularly in complex domains such as plant shoot structures where variation in genotype, background, and orientation is critical. Nonetheless, with sufficient input diversity and computational resources, our GAN-based pipeline could help extend training datasets in data-scarce domains such as plant phenotyping.

During the study, we worked with downsized images at resolutions of 512 $\times$ 512 and 1024 $\times$ 1024 to balance computational efficiency with image quality. Training the FastGAN model on 512 $\times$ 512 images required 5 h, while Training Pix2Pix on 512 $\times$ 512 images required 12 h, whereas on 1024 $\times$ 1024 images, it took 18 h. Higher-resolution images, while more computationally demanding, preserved the intricate details of plant structures more effectively, highlighting their potential for applications requiring fine-grained segmentation.

This approach demonstrates the feasibility of using downsized images for training under constrained hardware resources. However, applying this method to original high-resolution images (e.g., 3315 $\times$ 4462) would require significantly more computational resources, such as advanced GPUs, to handle the increased training time and memory demands. This insight is critical for practitioners considering similar models, emphasizing the importance of hardware capabilities in achieving optimal performance with high-resolution datasets. DatasetGAN [17] and BigDatasetGAN [18] leverage high-performance GPUs to generate pixel-wise annotations for multiple classes. DatasetGAN minimizes human effort by synthesizing labeled datasets, while BigDatasetGAN scales this approach to ImageNet-level datasets, enabling efficient and detailed annotations with minimal manual input. Compared with DatasetGAN and BigDatasetGAN—both of which require training large, class-agnostic GANs and then propagating fine-grained part labels from a small, manually annotated seed set—our FastGAN+Pix2Pix pipeline is lightweight (${\le }20$ GPU hours total) and directly produces paired RGB–mask data specific to plant shoots. Diffusion-based generators offer excellent texture fidelity but typically entail higher training and sampling costs (tens to hundreds of denoising steps) and do not natively output binary masks. By contrast, Pix2Pix learns an RGB$\Rightarrow$mask mapping and, combined with FastGAN’s species-specific synthesis, provides a practical route when labeled data and compute are limited.

One of the key successes of this research is the high accuracy achieved by Pix2Pix in annotating barley and *Arabidopsis* shoots. This shows the strong potential of Pix2Pix for automated plant phenotyping, where precise annotation is essential. The model’s ability to identify complex plant structures could significantly improve the efficiency and accuracy of analyzing phenotypic data. However, the results also show that the performance of both FastGAN and Pix2Pix still falls short of the accuracy achieved through manual annotation. This highlights the ongoing challenge of replicating the detailed judgment of human annotators for complex image datasets. Continued research and improvement in this area are needed to close the gap between automated and manual annotation methods. Our findings advocate for potential improvements in Pix2Pix’s performance through judicious calibration of hyperparameters, including mask dimensions, error metrics, cost functions, batch sizes, and learning rate configurations. Such alterations necessitate a deliberate equilibrium, as each modification could impart profound effects on the model’s operational efficacy.

While t-SNE embeddings provide qualitative insight into the diversity of generated shoot images, we also computed the Fréchet Inception Distance (FID) to quantify the similarity between real and synthetic distributions. Together with the observed spatial dispersion in the t-SNE plots, these results suggest that the GAN was able to avoid severe mode collapse across species. Nevertheless, we acknowledge that further improvements are possible. Future work will explore architectural enhancements such as StyleGAN, or training strategies like minibatch discrimination and diversity loss, to further increase sample variability and realism.

## Conclusion

In summary, our study demonstrates that GANs can be successfully adopted as a tool for generation of new ground truth images of greenhouse-grown plant shoots starting from a relatively small amount of reference data. For implementation of the GAN strategy, a two-stage pipeline consisting of FastGAN and Pix2Pix prediction models was used. While the GAN-derived labels approach manual accuracy (Dice 0.94–0.96), future work will evaluate training segmentation networks solely or partly on these synthetic pairs to quantify gains under annotation scarcity. Our findings demonstrate that the accuracy scores of 0.88$-$0.95 with respect to the Dice coefficient can be achieved crossover different plant types and optical setups. However, the GAN predictor still underperforms the manual image segmentation, which can be traced back to reduced variability of GAN generated features vs. real new data. Overall, we see great potential for automatically extending ground truth data in tasks of plant image analysis using generative AI models. However, future work should address limitations—such as the reduced variability of GAN-generated features relative to real data—evaluate newer generative paradigms, and embed plant-specific constraints to boost both realism and label fidelity.

## Supplementary Information

Supplementary file 1.

## Data Availability

No datasets were generated or analysed during the current study.
