# Supplementary material for: Automated generation of ground truth images of greenhouse-grown plant shoots using a GAN approach
Source: Plant Methods. 2025 Oct 4;21:126. doi: 10.1186/s13007-025-01441-1 (PMC12495773; doi:10.1186/s13007-025-01441-1)
Supplement: Supplementary file 1 [file 13007_2025_1441_MOESM1_ESM.pdf]

Supplementary Figures

Ullah et al. 'Automated Generation of Ground Truth Images of Greenhouse-grown Plant Shoots Using a GAN Approach', Plant Methods

August 8, 2025

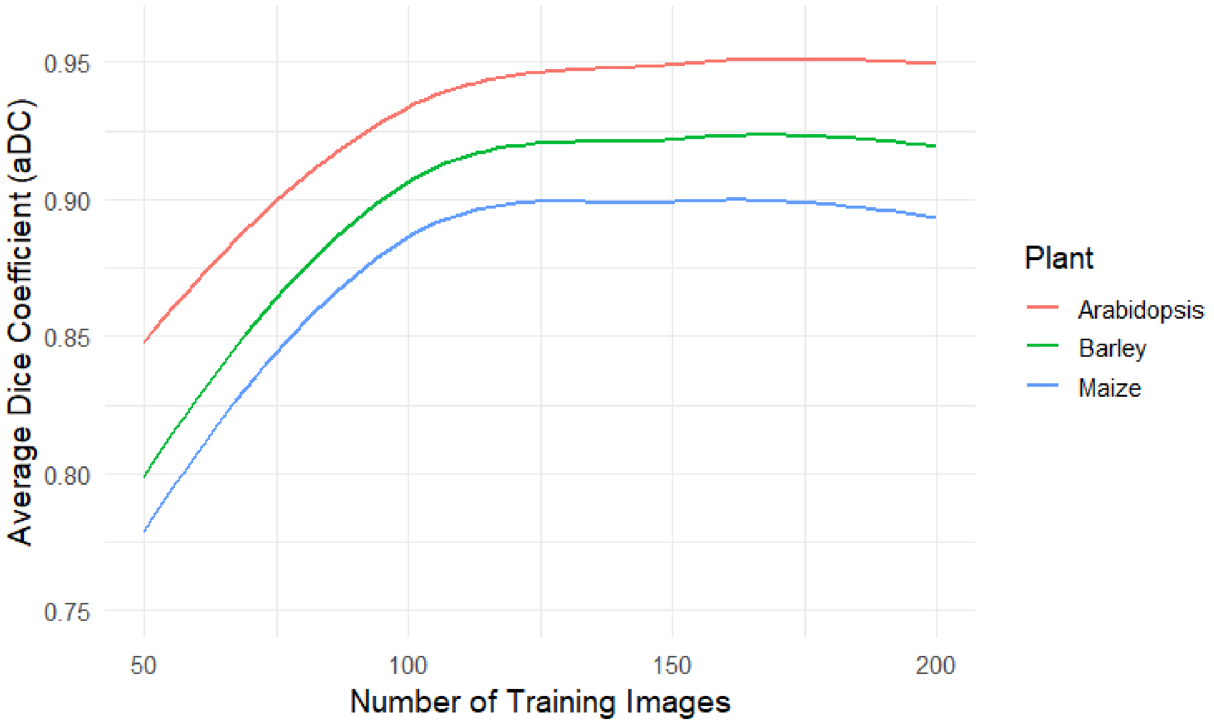

Figure 1: Average Dice Coefficient (aDC) of Pix2Pix trained on varying numbers of training images for *Arabidopsis*, barley, and maize plants.

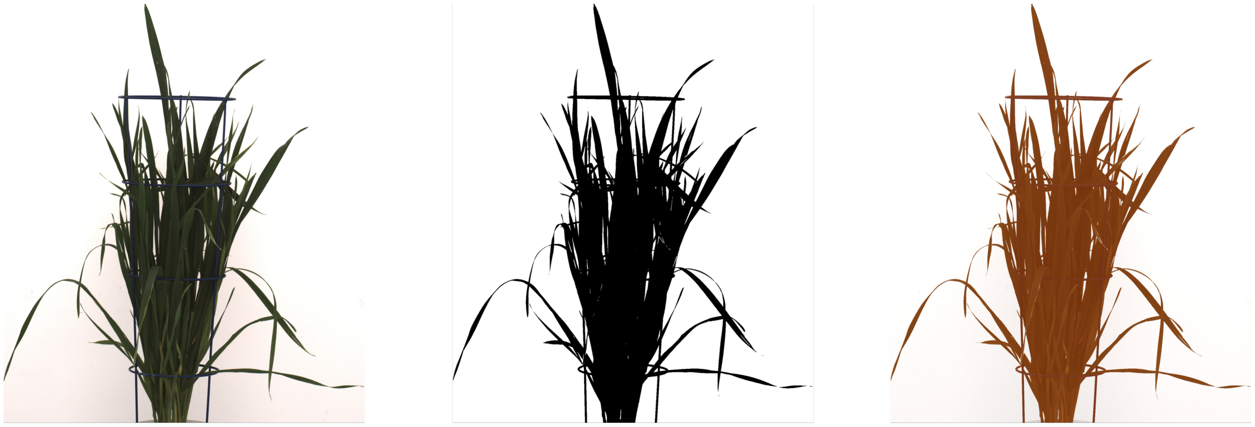

(a) (b) (c)

Figure 2: Example of Pix2Pix-based barley plant ground-truth. (a) Original RGB image. (b) Ground truth binary mask. (c) Overlay of Pix2Pix-predicted mask on the ground truth image.

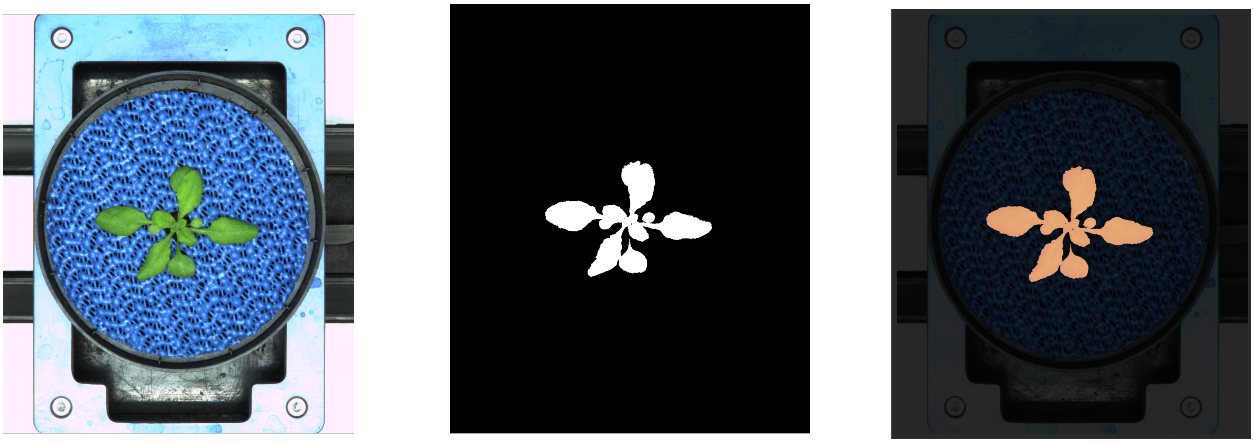

(a) (b) (c)

Figure 3: Example of Pix2Pix-based *Arabidopsis* plant ground-truth. (a) Original RGB image. (b) Ground truth binary mask. (c) Overlay of Pix2Pix-predicted mask on the ground truth image.

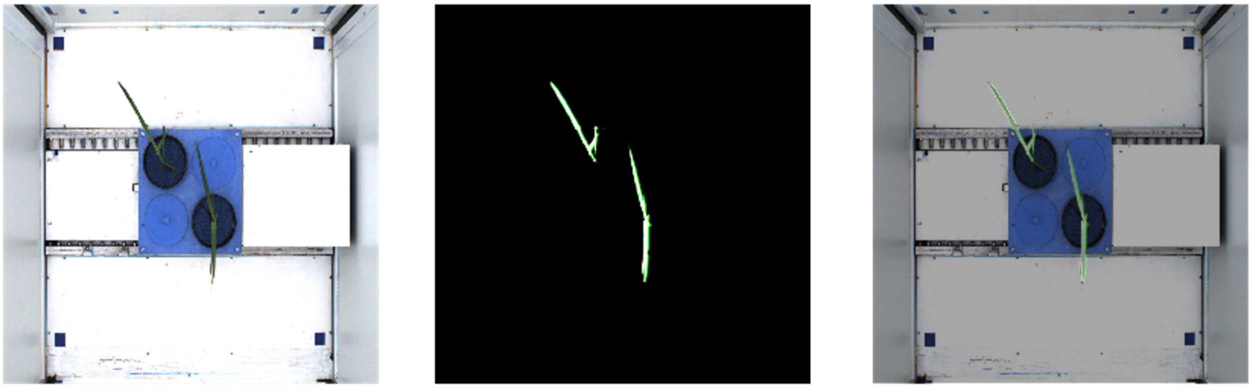

(a) (b) (c)

Figure 4: Example of Pix2Pix-based maize plant ground-truth. (a) Original RGB image. (b) Ground truth binary mask. (c) Overlay of Pix2Pix-predicted mask on the ground truth image.

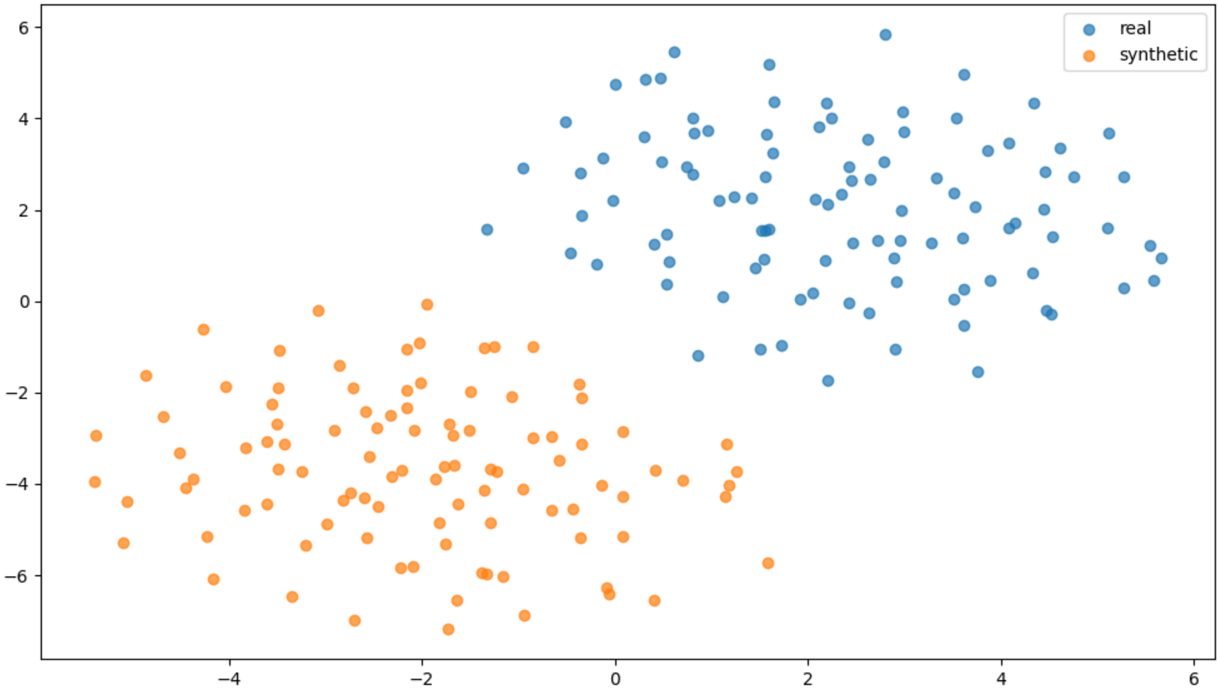

Figure 5: Visualization of t-SNE 1,2 components of feature embeddings extracted from real and GAN-generated RGB images of barley plants.

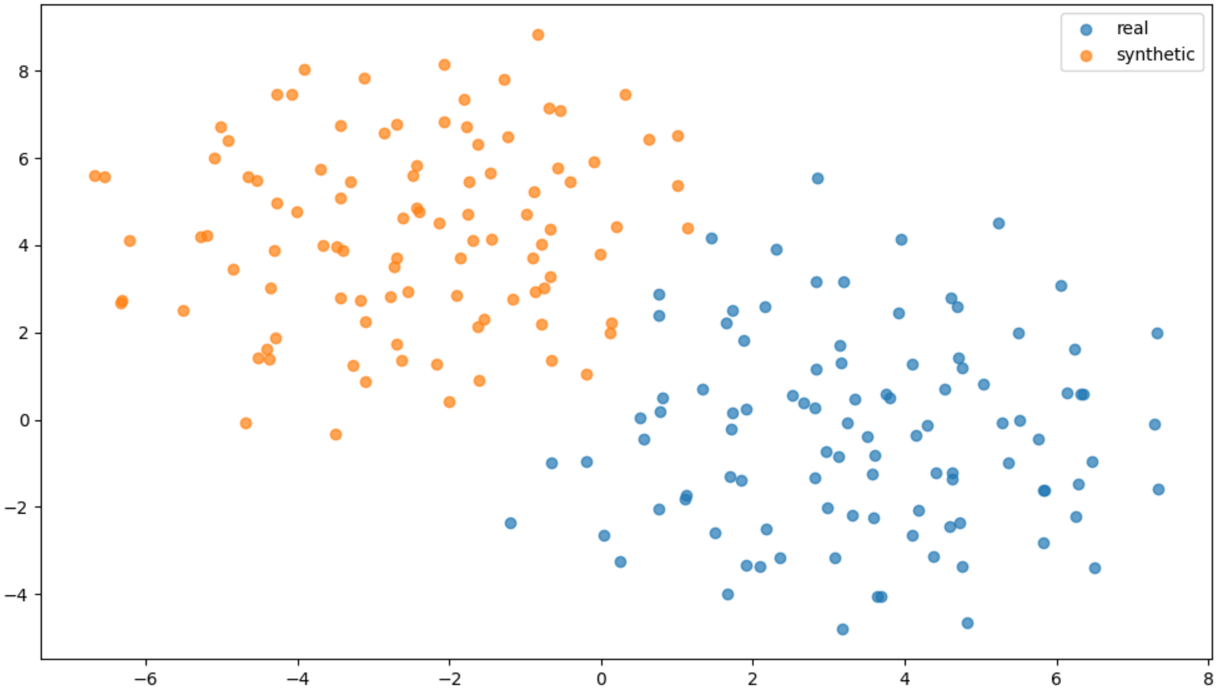

Figure 6: Visualization of t-SNE 1,2 components of feature embeddings extracted from real and GAN-generated RGB images of *Arabidopsis*.

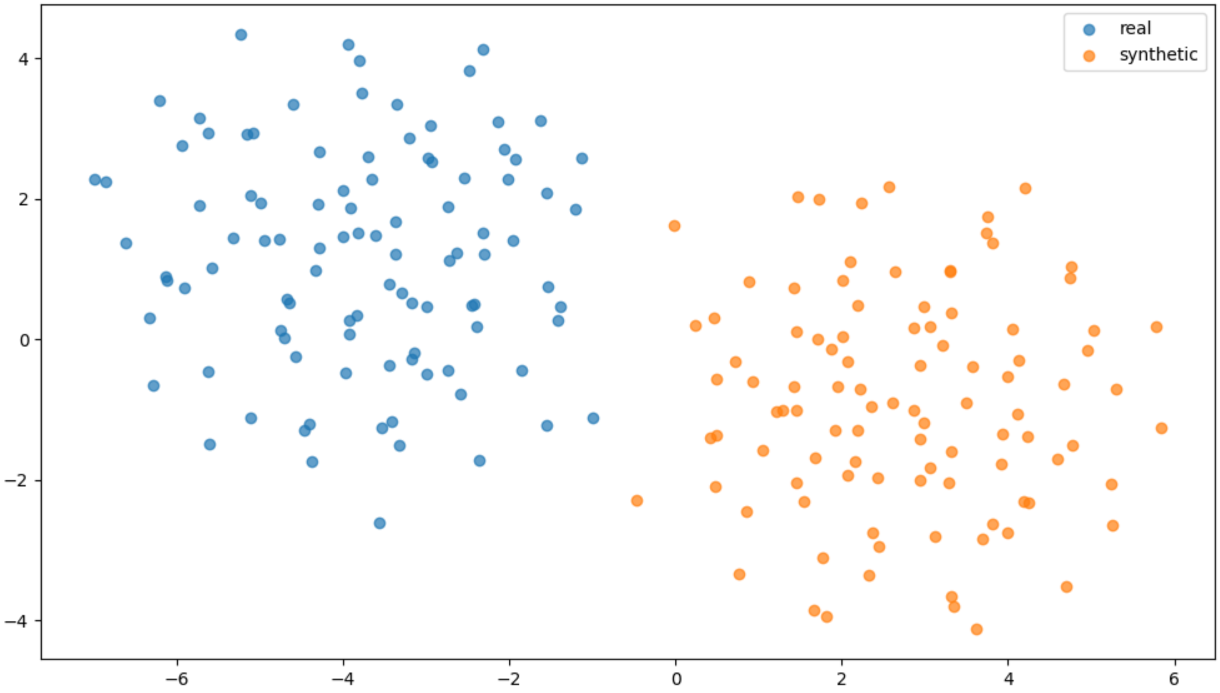

Figure 7: t-SNE visualization of feature embeddings extracted from real and GAN-generated RGB images of maize plants.
